# Identification of cancer risk lncRNAs and cancer risk pathways regulated by cancer risk lncRNAs based on genome sequencing data in human cancers

**DOI:** 10.1038/srep39294

**Published:** 2016-12-19

**Authors:** Yiran Li, Wan Li, Binhua Liang, Liansheng Li, Li Wang, Hao Huang, Shanshan Guo, Yahui Wang, Yuehan He, Lina Chen, Weiming He

**Affiliations:** 1 College of Bioinformatics Science and Technology, Harbin Medical University, Harbin, Hei Longjiang Province, Postal code: 150081, China; 2 National Microbology Laboratory, Public Health Agency of Canada, Winnipeg, Manitoba, Canada; 3 Institute of Opto-electronics, Harbin Institute of Technology, Harbin, Heilongjiang Province, Postal code: 150081, China

## Abstract

Cancer is a group of diseases involving abnormal cell growth with the potential to invade or spread to other parts of the body. The complexity of cancer can be reduced to a small number of underlying principles like cancer hallmarks which could govern the transformation of normal cells to cancer. Besides, the growth and metastasis of cancer often relate to combined effects of long non-coding RNAs (lncRNAs). Here, we performed comprehensive analysis for lncRNA expression profiles and clinical data of six types of human cancer patients from The Cancer Genome Atlas (TCGA), and identified six risk pathways and twenty three lncRNAs. In addition, twenty three cancer risk lncRNAs which were closely related to the occurrence or development of cancer had a good classification performance for samples of testing datasets of six cancer datasets. More important, these lncRNAs were able to separate samples in the entire cancer dataset into high-risk group and low-risk group with significantly different overall survival (OS), which was further validated in ten validation datasets. In our study, the robust and effective cancer biomarkers were obtained from cancer datasets which had information of normal-tumor samples. Overall, our research can provide a new perspective for the further study of clinical diagnosis and treatment of cancer.

Cancers are the consequence of a process of somatic mutation and break the balance controlled by gene expression programs and cellular networks that typically maintain intracellular homeostasis and prevent unnecessary expansion. Cancer hallmarks and important biological processes, such as cell growth, and cellular differentiation, were able to reveal neoplasia, growth and metastasis dissemination^1^. The identification of cancer related pathways was conducive to the comprehension of the potential mechanisms of tumorigenesis. Recent studies have linked multiple important biological pathways to the oncogenesis and progression of cancer, such as nuclear factor kB (NFkB) signaling pathway and Wnt/β-catenin signaling pathway^2^. Accurate regulation of NFkB activity is essential for physiological homeostasis, and was found that NFkB was over activated in a variety of cancers^3^. Wnt/β-catenin signaling pathway was suppressed by Frizzled-8, thus playing a crucial role in regulating numerous aspects of tumor development, including lung cancer^4^. The study on the regulation mechanism of pathways provides a framework for better understanding the diversity of cancers.

In the past, the majority of studies for the mechanisms of carcinogenesis mainly focused on protein-coding genes. Recently, abnormal expressions of long noncoding RNAs (lncRNAs) identified by the next-generation sequencing technologies are related to different types of cancer. The integration of lncRNAs expression profiles offers an impactful approach to study the lncRNA regulation mechanism of cancer-related pathways^5^. LncRNAs are non-protein-coding RNA molecules which could regulate gene expression at diverse levels, containing histone modification, transcription, and/or posttranscriptional regulation. They act as activators, guides, or scaffolds for proteins, DNA and RNA, and would be possible drivers of carcinogenesis biology and work as clinical biomarkers.

Emerging investigations have found that lncRNAs could regulate pathways to act as a main contributor to carcinogenesis. In previous studies, the individual lncRNA contributions to a single pathway in a specific cancer were taken into account from the experimental perspective. For example, lncRNA CCHE1 indicated poor prognosis in hepatocellular carcinoma (HCC) by activating the ERK/MAPK pathway to promote tumorigenesis^6^. Lnc_bc060912 whose expression increased in human lung and other tumors and affected cell apoptosis via PARP1 and NPM1 which were two DNA damage repair protein^7^.

However, the joint effect of common lncRNAs contributing to a complex cancer was not assured by previous studies. In fact, one pathway could be regulated by multiple lncRNAs in various cancers, and one lncRNA could regulate different pathways associated with different cancers. For example, lncRNAs AK126698, CASC11 and UCA1 regulated the Wnt/β-catenin pathway in non-small cell lung cancer^4^, colorectal cancer^8^ and oral squamous cell carcinoma^9^, respectively^10^. Moreover, many studies have shown that a number of lncRNAs were involved in the p53 pathway. For example, tumor suppressor response lncRNAs LOC572558 and MT1JP regulated the p53 signaling pathway in bladder cancer and other cancers, respectively^11^. In addition, functional lncRNAs used in this study were annotated to biochemical pathways by LncRNA2Function^12^, which considered all lncRNAs in the pathways as equal, rather than discovered their potential relationship with human cancers.

Thus, this study focused on the joint effect of common lncRNAs which have not studied and reported in regulating common cancer related pathways in pan-cancers. Based on lncRNA expression profiles and clinical data of human cancer patients from TCGA, we examined differentially expressed lncRNAs and mRNAs for multiple cancer datasets. Above all, cancer risk pathways and lncRNAs were identified using Wilcoxon signed-rank test and permutation test which were closely associated with not only prognosis-related functions, but also survival of cancer patients. The robustness of these lncRNAs was verified by independent profiles from another platform. By investigating lncRNAs and their regulating pathways in cancer patients, our study would provide insights into the oncogenesis and progression of cancers.

## Results

### Abnormal mRNAs and lncRNAs for cancers

For each type of human cancer, DE protein-coding genes and lncRNAs were identified through t-test, controlling False Discovery Rate (FDR) at 5%. Through calculated reads per kilo bases per million reads (RPKM) values for the lncRNA or mRNA in human normal and cancer samples from TCGA, 19901 mRNAs and 14373 lncRNAs expression were recognized.

### Cancer risk pathways

Cancer associated candidate pathways were defined as significant pathways using the Wilcoxon signed-rank test after 1000 permutation tests (FDR < 0.05) in each cancer dataset. There were 419, 835, 1048, 398, 1457 and 677 biochemical pathways which were identified as cancer associated candidate pathways in BLCA, BRCA, KICH, KIRC, LUAD and PRAD, respectively. These 6 cancer risk pathways were shared among six cancer datasets: “Anaphase-promoting complex/cyclosome (APC/C) -mediated degradation of cell cycle proteins”, “Cyclin B2 mediated events”, “PLK1 signaling events”, “Mitotic Prometaphase”, “Beta defensins” and “Defensins” pathways (Supplementary Table 1) and were thus termed as common cancer risk pathways.

APC/C-mediated degradation of cell cycle proteins pathway has been confirmed to involve in colorectal cancer^13^,^14^. Due to a better understanding of APC/C which involved in mitosis and established a stable G1 phase, the understanding of DNA damage or perturbation of the normal cell cycle had been greatly improved^15^. In many renal cell carcinomas, prostate cancers, basal cell carcinomas and oral squamous cell carcinomas (OSCC), Defensins pathway^16^ might participate in the regulation of oncogenesis and changed the expression of β-defensins^17^. A new study had indicated that β-defensins could mediate specific antineoplastic immunity and enhance antineoplastic consequences, in which also suggested that β-defensins deserved further examination as potential neoplastic immunotherapy immunogenes^18^. The over-expression of β-defensins in cancers was found by Semple F^17^. In addition, the rest of the cancer risk pathway: Mitotic Prometaphase pathway and Cyclin B2 mediated events pathway, which usually show abnormal activities and will result in poor prognosis^19^ in patients of breast cancer.

### Cancer risk lncRNAs

**Figure 1:**
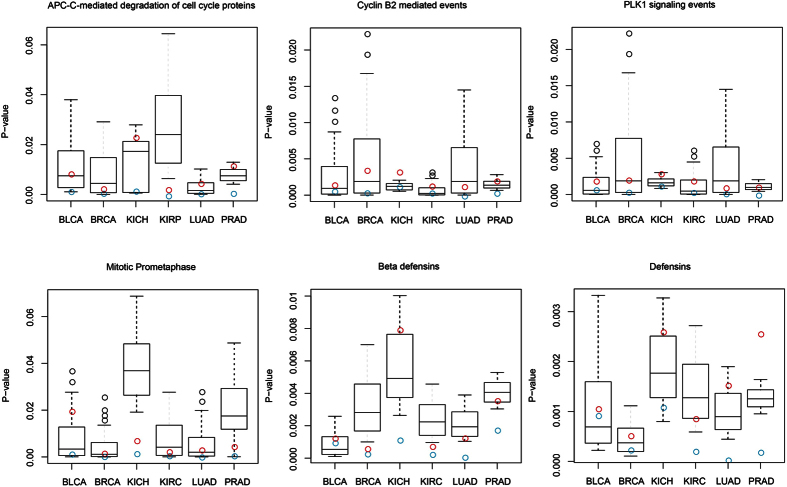
The 3% of the smallest permutation p values of 23 cancer risk lncRNAs. (**A**) The 3% of the smallest permutation p values in BLCA. (**B**) BRCA. (**C**) KICH. (**D**) KIRC. (**E**) LUAD. (**F**) PRAD. In the boundary of the 3% of the smallest permutation p values in six cancer datasets, the blue open circles and red open circles are the real p value of 23 cancer risk lncRNAs and the real p value of entire lncRNAs in six cancer risk pathways, respectively. The p values of 23 cancer risk lncRNAs were smaller than the p value of entire lncRNAs for each cancer risk pathway.

As lncRNAs may be pivotal in many biochemical pathways^20^, we explored whether cancer risk lncRNAs from different cancer datasets display a similar regulation pattern with their cancer risk pathways or not. A total of 23 lncRNAs which involved in the regulation of cancer risk pathways were identified as cancer risk lncRNAs in the six cancer datasets. The performance of identifying cancer risk pathways of 23 cancer risk lncRNAs was evaluated in each cancer dataset for 1000 permutation tests, respectively (Figure S1). Most cancers showed no significance of permutation p values for their corresponding risk pathways (Wilcoxon signed-rank test >0.05). Then, 3% of the smallest permutation p values were selected as shown in Fig. 1. No matter the real p value of 23 cancer risk lncRNAs or the real p value of entire lncRNAs, both of them were remained in the boundary of the 3% of the smallest permutation p values. In addition, the p values of 23 cancer risk lncRNAs were smaller than the p value of entire lncRNAs for each cancer risk pathway. During the process of using lncRNAs to identify pathways, the p values of 23 cancer risk lncRNAs were much smaller than the p values of the whole lncRNAs within pathways. The result suggested that 23 cancer risk lncRNAs having better cancer risk pathway detection efficacy than the whole lncRNAs.

A number of studies have indicated that these 23 cancer risk lncRNAs were closely related to the occurrence or development of cancer. For example, Hassan M suggested that AC005076 might be a functional lncRNA to intervene apoptosis, and might be associated with cancer therapies clinically^21^. LINC00654 and STK4-AS1 were served as prognostic lncRNAs for cancers like breast or prostate^22^. Particularly the lncRNA RP4-612B15 showed delicate changes of genome in mantle cell lymphomas (MCL), a subset of B-cell non-Hodgkin’s lymphomas, and had been acknowledged as candidate neoplasia functional lncRNA, which suggested its potential as suppressor lncRNA^23^. Besides, viral infection is related to the development of lots of cancers, such as cervical cancer^24^ and liver cancer. NRAV, which expressed in numerous tissues, had been considered as a pivotal contributor of antiviral innate immunity. NRAV could be associated with the pathogenesis of cancers caused by viruses^25^.

### Evaluation of the performance of cancer risk lncRNAs

#### Functional annotation of cancer risk pathways and risk lncRNAs

**Figure 2:**
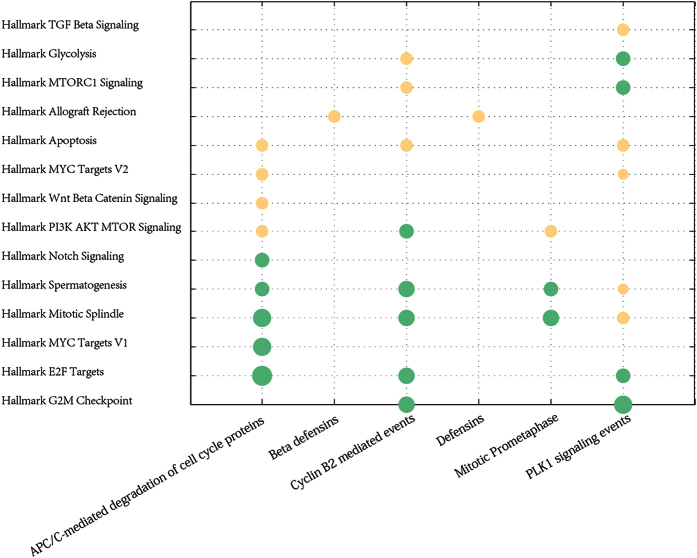
Enrichment analysis delineates cancer risk pathways and cancer hallmark-associated GO terms. R statistical software was used for visualization of the enrichment results. The bubble size indicates the p value of hypergeometric test between each term and each cancer risk pathway, and different color corresponds to different FDRs. The darker of the color, the smaller of the FDR.

Abnormal pathways generally take place in human cancers and usually cause insensitive treatment of cancer. Even though the biological characteristics of cancer are extremely complicated and which can be reduced and expressed by a small number of cancer hallmark-associated GO terms which can lead to tumor growth and metastasis dissemination^26^. These hallmark-associated GO terms provide a framework for comprehending the noteworthy multiplicity of cancers. To reveal the cancer risk pathways regulated by 23 risk lncRNAs that may have functions in tumor-promoting or suppressing, lots of cancer hallmark-associated GO terms were enriched in cancer risk pathways (hypergeometric test, FDR < 0.05, Fig. 2).

It was demonstrated that each cancer risk pathway enriched at least one hallmark-associated GO terms of cancers. In total, six cancer risk pathways were enriched in fourteen hallmark-associated GO terms of cancers. Among them, two cancer risk pathway “APC/C-mediated degradation of cell cycle proteins” and “Defensins” were enriched with nine hallmark-associated GO terms, respectively. “PLK1 signaling events” enriched with eight hallmark-associated GO terms and “Beta defensins” enriched with three hallmark-associated GO terms.

It was shown that some hallmark-associated GO terms were shared by several cancer risk pathways. Especially, four of six cancer risk pathways: “APC-C-mediated degradation of cell cycle proteins pathway”, “Cyclin B2 mediated events pathway”, ”PLK1 signaling events pathway” and “Mitotic Prometaphase pathway” were enriched in “Hallmark mitotic spindle” and “Hallmark spermatogenesis”, these two hallmark-associated GO terms were important for cell division^27^ and development^28^. Hong tao Yu^29^ pays attention to the corrected positioning of the mitotic spindle. Due to sister-chromatid not accurately attached to the mitotic spindle, the spindle checkpoint facilitates the assembly of checkpoint protein complexus that restrain the action of APC/C, resulting in the steadiness of securin, protection of sister-chromatid cohesion, and a delay in the beginning of anaphase^30^. The “Hallmark apoptosis”, a common feature of cancers, was enriched with APC/C-mediated degradation of cell cycle protein, Cyclin B2 mediated events and PLK1 signaling events, highlighting their roles in the development of cancers. In addition, these three cancer risk pathways were also enriched in “Hallmark E2F targets” (Fig. 2). E2F transcription factors acts a functional role in cell proliferation^31^, and is deregulated pRB pathway, which is a very recurrent occurrence in human cancer, suggesting these three risk pathways might be carcinogenesis traits of cancers. In addition, “Cyclin B2 mediated events” and “Mitotic Prometaphase” were enriched with “Hallmark allograft rejection”, respectively. Meanwhile, these cancer risk pathways were enriched in core hallmark-associated GO terms related to cancer, such as, apoptosis, mitotic spindle and glycolysis, suggesting risk lncRNAs will impact greatly on our knowledge and understanding of cancer risk pathways in human cancers.

#### The classification ability of the identified cancer risk lncRNAs in six internal test datasets

**Figure 3:**
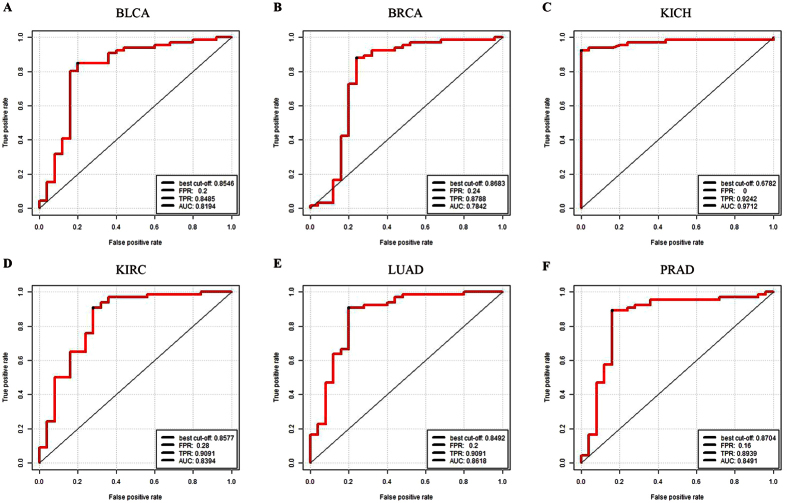
Five-fold cross-validation for the cancer risk lncRNAs in six cancer datasets. (**A**) Five-fold cross-validation for BLCA. (**B**) BRCA. (**C**) KICH. (**D**) KIRC. (**E**) LUAD. (**F**) PRAD. It is distinct that 23 cancer risk lncRNAs are robust and sensitive in distinguishing normal and tumor samples in six cancer datasets. FPR: False Positive Rate. TPR: True Positive Rate. AUC: Area Under the Curve.

With expression values of 23 cancer risk lncRNAs obtaining from the training dataset as entered features, the SVM classifier was used to discriminate cancer patients and normal samples in six internal test datasets. Based on AUC values, five-fold cross-validation method was used to assess the classification ability between normal and cancer samples, as described in the Methods section. It was shown that 23 cancer risk lncRNAs had a good distinguish performance in six internal test datasets through the cross-validation approach. The average AUC values of 1000 permutation tests for 6 cancer datasets were calculated, which were 0.8194, 0.7843, 0.9712, 0.8339, 0.8618 and 0.8491, respectively, (Fig. 3A–F), which indicated a high classification performance. In the six internal test datasets, it was suggested that the 23 cancer risk lncRNAs within six cancer risk pathways could be used as the classification features to recognize normal samples and cancer samples.

#### The prognosis of cancer risk lncRNAs

**Figure 4:**
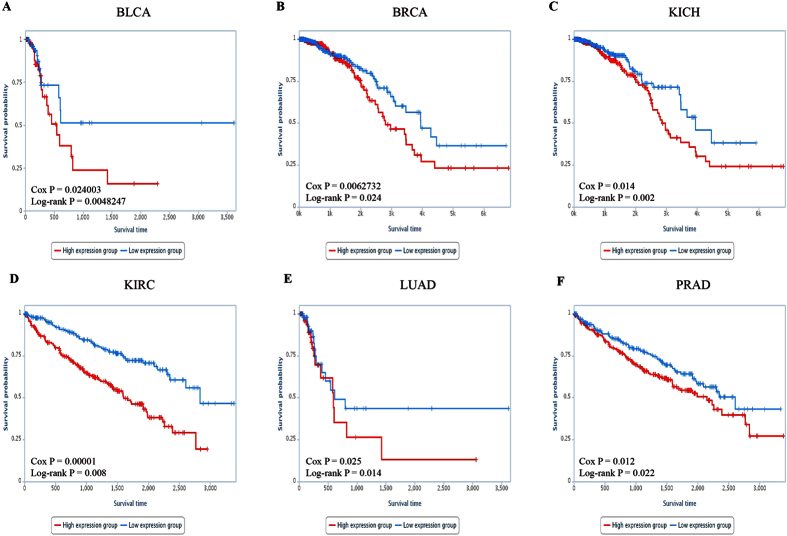
Survival ananlysis of six cancer datasets. Kaplan-Meier curve for overall survival of two samples groups with higher (top 50%) or lower (bottom 50%) expression of 23 cancer risk lncRNAs in (**A**) BLCA. (**B**) BRCA. (**C**) KICH. (**D**) KIRC. (**E**) LUAD. (**F**) PRAD. The blue curve indicates higher expression group and the red curve indicates lower expression group.

Kaplan-Meier curves for the two groups (high-risk group or low-risk group) within six cancer datasets were shown in Fig. 4, representing significant difference between high-risk group and low-risk group in OS. The significant p values of Cox regression analysis and log-rank test observed in six cancer datasets for each lncRNA were displayed in Supplementary Table 2.

#### The classification ability of the identified risk lncRNAs in validation datasets

**Figure 5:**
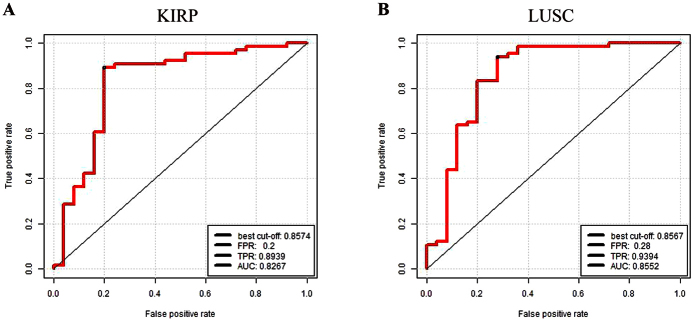
Five-fold cross-validation for the cancer risk lncRNAs in two cancer datasets. (**A**) Five-fold cross-validation for KIRP. (**B**) LUSC. FPR: False Positive Rate. TPR: True Positive Rate. AUC: Area Under the Curve.

Cancer is a group of diseases involving abnormal cell growth with the potential to invade or spread to other parts of the body. However, the growth and metastasis of cancer often relate to combined effects of lncRNAs. Some prognostic markers have been identified, but the robustness of these prognostic markers was not sufficient. Thus, the robustness of these lncRNAs was verified by eight independent profiles from another platform. The validation datasets contained two independent cancer datasets (KIRP and LUSC) from TCGA IlluminaHiSeq RNASeqV2 data, other eight additional independent validation datasets (BLCA, BRCA, HNSC, KIRC, LIHC, LUAD, LUSC and UCEC) from TCGA IlluminaHiSeq RNASeq data. The AUC values of KIRP and LUSC were 0.8267 and 0.8552 (Fig. 5A,B), which demonstrated high classification performance.

**Figure 6:**
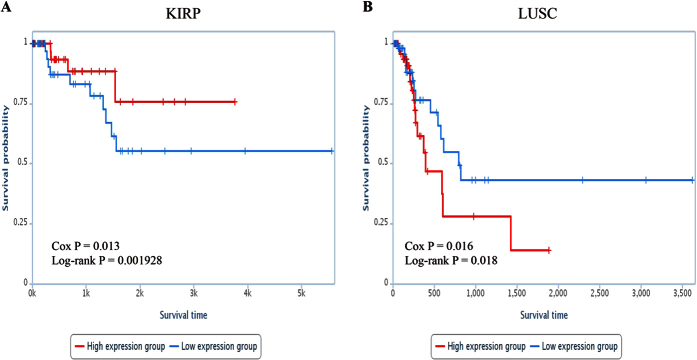
Survival ananlysis of KIRP and LUSC. Kaplan-Meier curve for overall survival of two samples groups with higher (top 50%) or lower (bottom 50%) expression of 23 cancer risk lncRNAs in (**A**) KIRP. (**B**) LUSC. The blue curve indicates higher expression group and the red curve indicates lower expression group.

The AUC values of eight additional independent validation datasets were 0.8079, 0.8661, 0.8655, 0.8145, 0.8837, 0.9751, 0.8437, 0.9424 (Fig. 6A–H), indicating high classification power with eight independent datasets. This also suggested that 23 cancer risk lncRNAs which obtained from performing Wilcoxon log-rank test to the six cancer risk pathways had a good classification performance to distinguish normal and tumor samples in other types of cancer datasets.

#### The prognosis of cancer risk lncRNAs in two validation datasets

**Figure 7:**
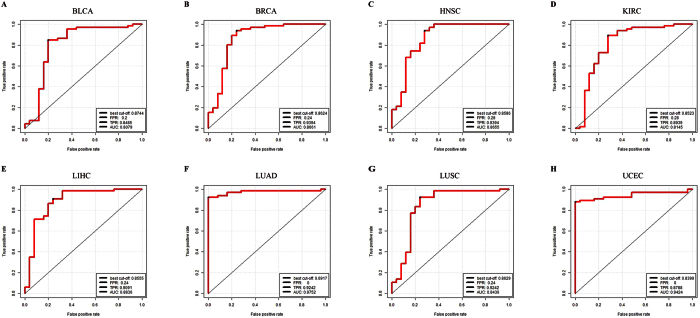
Five-fold cross-validation for the cancer risk lncRNAs in eight cancer datasets. (**A**) Five-fold cross-validation for BLCA. (**B**) BRCA. (**C**) HNSC. (**D**) KIRC. (**E**) LIHC. (**F**) LUAD. (**G**) LUSC (**H**) UCEC. FPR: False Positive Rate. TPR: True Positive Rate. AUC: Area Under the Curve.

In another two independent cancer datasets (KIRP and LUSC) which were also based on the IlluminaHiSeq platform, we used expression values of 23 cancer risk lncRNAs and clinical information of samples to conduct survival analysis aiming to further validate the robustness of the 23 cancer risk lncRNAs. The significant p values of Cox regression analysis observed in two cancer datasets and the Kaplan-Meier curves were displayed in Fig. 7, respectively. Samples of LUSC dataset assigned into high-risk group tended to have shorter OS than those in the low-risk group (log-rank test p = 0.018). On the contrary, high-risk group tended to have longer OS than those in the low-risk group (log-rank test p = 0.002) in KIRP dataset.

### The stability and robustness of our approach

To show the robustness of the predictors, re-sampling statistics are required, because Li *et al*. showed that cancer heterogeneity often prevents cancer biomarkers to be robust^32^. It is desirable to discuss different predictors representing different cancer risk pathways and cancer risk lncRNAs could be combined and complementary for better predictions^33^. We used the 1000 times leave one out cross-validation with a Support Vector Machine (SVM) method for both the discovery dataset and the validation dataset. It was shown that 23 cancer risk lncRNAs had a good distinguish performance in six internal discovery datasets through the 1000 times leave one out cross-validation approach. The average AUC values of 1000 permutation tests for 6 cancer datasets were calculated, which were both 0.9527, 0.9873, 0.9894, 0.9682, 0.9691 and 0.8867, respectively, (Supplementary Figure 2), which indicated a high classification performance. The AUC values of KIRP and LUSC were 0.9812 and 1 (Supplementary Figure 3A and B), which demonstrated high classification performance. The AUC values of eight additional independent validation datasets were 0.8079, 0.8412, 0.8455, 0.8491, 0.8533, 0.8518, 0.8836, 0.9476, 0.9521 (Supplementary Figure 4A–H), indicating high classification power with eight independent datasets. In both the discovery dataset and the validation dataset, it was suggested that the 23 cancer risk lncRNAs within six cancer risk pathways could be used as the classification features to recognize normal samples and cancer samples. In order to confirm the high classification performance and stability of the cancer risk lncRNAs identified by our approach, we used the naive Bayes to evaluate the classification performance taking the cancer risk lncRNAs expression values as the classification features in the same way. A naive Bayes classifier is a simple probabilistic classifier based on applying Bayes theorem with strong (naive) independent assumptions. The AUC values of the discovery and the validation dataset were both above 0.840 (Supplementary Figures S3, S5 and S6). The cancer risk lncRNAs had a good classification performance and stability by not only the SVM methods (Supplementary Figures 2–4) but also by naive Bayes (Supplementary Figures S3, S5 and S6).

## Discussion

At present, the research of lncRNAs in different aspects and frontiers is increasing. However, understanding the best way of lncRNAs in regulating pathways for giving insight into underlying mechanism and development of cancer is still in its infancy. In this study, we selected RNASeq data of eleven human cancers from publicly available repositories and calculated expression change (Δe) for each lncRNA while using Wilcoxon signed-rank test of Δe for all lncRNAs/genes within a pathway to detect the cancer risk pathway and cancer risk lncRNA. Coupled with clinical information, these cancer risk lncRNAs were used to conduct survival analysis. For six cancer datasets, 6 cancer risk pathways and 23 cancer risk lncRNAs were identified. Kaplan-Meier curves by 23 cancer risk lncRNAs within six cancer datasets demonstrated a significant difference in OS between high-risk group and low-risk group.

Pathways could be major contributors for cancer, and lncRNAs play key roles in cancer occurrence^34^. Here, six cancer risk pathways (“APC-C-mediated degradation of cell cycle proteins”, “Cyclin B2 mediated events”, “PLK1 signaling events”, “Mitotic Prometaphase”, “Beta defensins” and “Defensins”) in six human cancers were identified based on p values of Wilcoxon signed-rank test and were evaluated through five-fold cross-validation approach. These cancer risk pathways were not only significantly enriched functional specificity with hallmark classes of human cancer, but also widely confirmed associated with cancers by literatures. In addition, significant cancer risk pathways identified by lncRNAs for six cancers could not be found by mRNAs using the same approach. In brief, these six cancer risk pathways would be regulated by lncRNAs in the carcinogenesis.

Notably, 23 common lncRNAs within six cancer risk pathways were considered as cancer risk lncRNAs. In the identification process of cancer risk pathways, the significance of 23 cancer risk lncRNAs was higher than entire lncRNAs (Fig. 1). In other words, 23 cancer risk lncRNAs could represent entire lncRNAs while identifying cancer risk pathways. In addition, SVM was adopted with 23 cancer risk lncRNAs expression values as classification features to distinguish normal and tumor samples. And then classification performance (Fig. 3) was estimated by a receiver operating characteristic (ROC) curve to further evaluate the relationship between cancers and these 23 cancer risk lncRNAs. These cancer risk lncRNAs not only had a good classification result in the six test datasets, but also achieved good results in two validation datasets and eight additional independent validation datasets. (Figs 4 and 7) It suggested that these risk lncRNAs could be new and potential biomarkers for human cancers.

Furthermore, the weighted voting classification algorithm was adopted to investigate the classification ability of 23 cancer risk lncRNAs for normal and tumor samples. Firstly 23 risk lncRNAs were ranked according to signal-to-noise metric. Then the average number of misclassified patients of the 5-fold cross-validation in 1000 permutations was calculated when increasing numbers of top ranked predictive lncRNAs (Fig. 1). As a result, one specific lncRNA was identified as optimal cancer-related lncRNA for each cancer dataset respectively (Supplementary Table 3). Four lncRNAs were found to be the optimal cancer-related lncRNA for six cancer datasets. Especially, NRAV as a key regulator of antiviral innate immunity^25^ had been identified in three cancers (BLCA, KICH and KIRP). RP4-612B15 showing subtle genomic alterations in mantle cell lymphomas^23^ had been found in BRCA and PRAD. In summary, the result above suggested that 23 cancer risk lncRNAs had the potential to be candidate tumor lncRNAs.

In addition, 23 cancer risk lncRNAs had a good classification performance for samples, and were able to separate samples in the entire cancer dataset into two groups with significantly different OS. With 23 cancer risk lncRNAs for each cancer dataset, the Kaplan-Meier analysis for OS demonstrated a significant difference between the groups predicted to be high expression group or low expression group (p = 3.44e-15, log-rank test; Fig. 4). The Cox regression p values and Log rank p values were not only significantly associated with OS in the six test datasets, but also in two validation datasets and eight additional independent validation datasets (Supplementary Table 2). Therefore, 23 cancer risk lncRNAs within six cancer risk pathways could identify the survival difference between two groups of samples in sixteen cancer datasets, and these risk lncRNAs could be potential prognostic biomarkers for human cancers with stability and robustness.

We apply a relatively novel perspective way to identify cancer risk pathways and potential lncRNAs of human cancers. Indeed, focusing on the combination of pathways and lncRNAs could help reveal many potential lncRNAs which capable of taking effect in the occurrence and development process of cancer. It was worth noting that 23 cancer risk lncRNAs identified by our approach were obtained from diverse cancer tissues, while lncRNAs were generally considered to be associated with specific tissues^35^. The cancer risk pathways regulated by these lncRNAs were also the cardinal factor in the progression and metastasis of various carcinomas^36^. Thus, our findings highlighted the cancer common features shared by pan-cancer.

In our study, the robust and effective cancer biomarkers were obtained from cancer datasets which had information of normal-tumor samples. For cancers without normal sample information, our approach could also be used to identify biomarkers related to molecular subtypes of cancers in their clinical outcome. Overall, our research can provide a new perspective for the further study of clinical diagnosis and treatment of cancer.

## Methods

### Materials

#### TCGA datasets and clinical information of cancer patients

**Figure 8:**
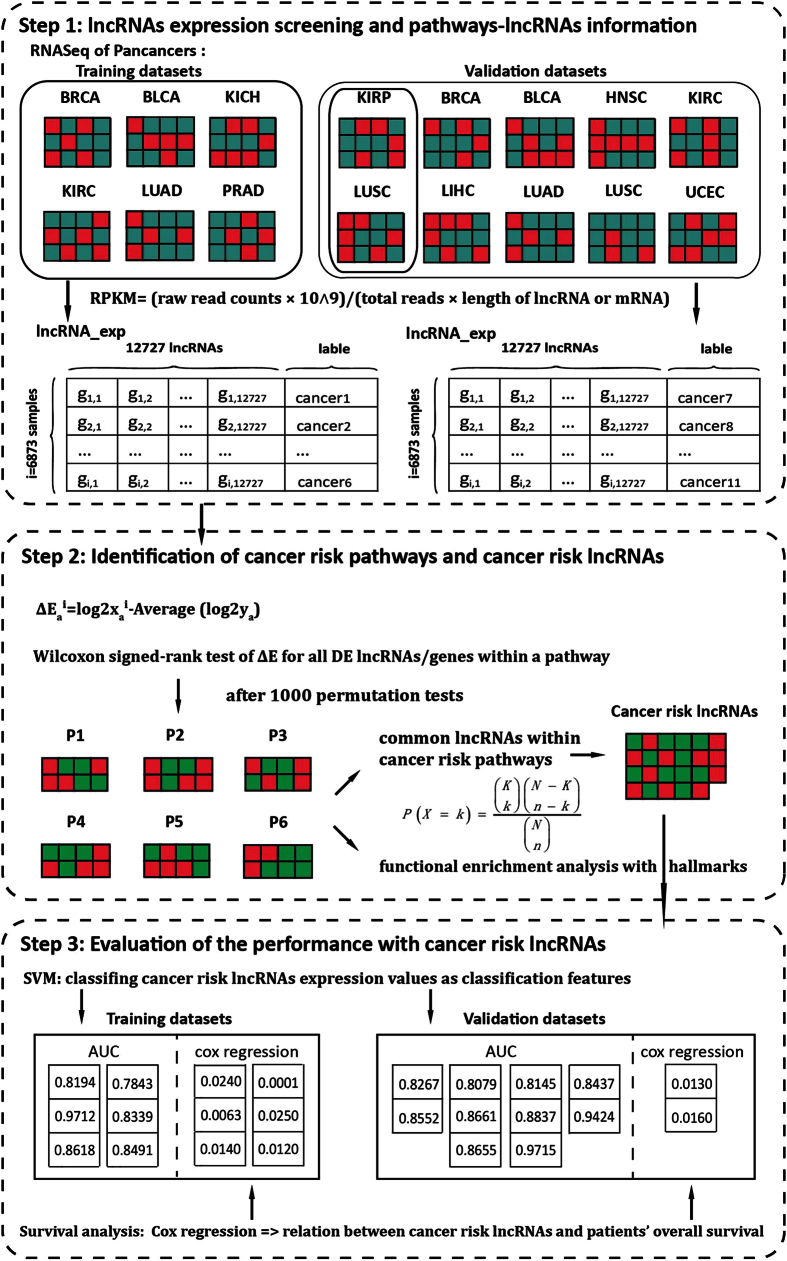
The workflow of this study. Step one, lncRNAs expression screening and pathways-lncRNA information. First, six cancer datasets were used as training datasets and ten cancer datasets were used as validation datasets. Then RPKM values were calculated for each lncRNA or mRNA. Step two, identification of cancer risk pathways and cancer risk lncRNAs according to Wilcoxon signed-rank test of Δe for all DE lncRNAs/genes within a pathway. Step three, Evaluation of the performance with cancer risk lncRNAs based on SVM and survival analysis.

Illumina RNA (IlluminaHiSeq RNASeqV2 and IlluminaHiSeq RNASeq) sequencing data for eleven types of human cancers which contained cancer and normal samples with clinical information (see Supplementary Table 4), were obtained from TCGA through Data Portal^37^. Raw read counts of each exon were originated from annotated exon quantification files offered by TCGA level3 datasets. Annotation of exons mapping to lncRNA or mRNA was downloaded from GENCODE^38^. Cancer patients and tumor features are detailed in Supplementary Table 5. The whole workflow for this study was shown in Fig. 8.

#### LncRNA and mRNA expression profiles across cancers

Separately for each cancer dataset, the expression level of lncRNA or mRNA was calculated through computing reads per kilo bases per million reads (RPKM) values for the lncRNA or mRNA:


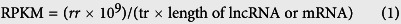


where rr means sum of raw read counts in all exons mapped within a lncRNA or mRNA; tr equal sum of raw read counts computed for all exons of every sample. We discarded lncRNAs whose RPKM expression values were 0 in all samples to filter out lncRNAs which were not expressed across samples in sequencing data. The lncRNAs with more than 30% missing values in all samples were also removed from this study. All the expression values of lncRNAs and mRNAs were log2 transformed. LncRNAs RPKM expression values of 0 were changed to 0.05 to allow log transformation.

#### Hallmark-associated GO terms and pathway information

A collection of Gene Ontology (GO) terms that were associated to the hallmark-associated GO terms of cancer were derived from a previous study^39^. Genes annotated to fifty hallmark-related GO terms were downloaded from MsigDB V5.1^40^. Moreover, the pathway data obtained from Consensus Path Data Base (CPDB)^41^ were used for the subsequent analysis.

## Methods

### Identifying mRNAs and lncRNAs related to cancers

For each type of human cancer, we identified differentially expressed (DE) protein-coding genes and lncRNAs by comparing expression values of cancer samples to those of normal controls. Two-tailed T-test was used to identify the differentially expressed lncRNAs and protein-coding genes. Multiple hypothesis testing using Benjamini and Hochberg’s method^42^ was used to adjust the differential expression p-values, controlling False Discovery Rate (FDR) at 5%. Protein-coding genes and lncRNAs with FDR<0.05 were deemed as differentially expressed.

### Identifying significant cancer risk pathways

To identify risk pathways associated with tumor patients, the Wilcoxon signed-rank test was used to measure pathways with significant expression changes. In tumor sample n and the corresponding normal samples, we calculated expression change for every DE lncRNA/gene m,


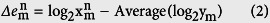


where x_m_^n^ is the expression value of DE lncRNA/gene m in tumor sample n, and y_m_ is the expression in the q matching normal samples (y^1^, y^2^, …, y^q^). Then, the significance of a pathway of a cancer dataset was evaluated by Wilcoxon signed-rank test for Δe, controlling FDR at 5%.

Using 1000 permutation tests as follows to evaluate the significance of the expression changes for each pathway in a cancer dataset: (i) tumor and normal samples of each cancer dataset were divided into five non-overlapping parts, respectively. (ii) four of five parts were included in training dataset, and the remaining samples were used for validation in further analysis. (iii) lncRNAs/genes within a pathway were randomly selected, and equal number of DE lncRNAs/genes was maintained for each permutation test. (iv) the significance of Wilcoxon signed-rank test for each pathway was re-calculated. P value was evaluated as the percent of insignificance in 1000 permutation tests. A significant pathway (FDR < 0.05) was considered as cancer associated candidate pathway. Here, cancer risk pathways were termed as common cancer associated candidate pathways of six cancer datasets.

### Identification of cancer risk lncRNAs

Common lncRNAs of six human cancers within cancer risk pathways were considered as cancer risk lncRNAs. To evaluate the significance of the pathway in tumor samples of each specific cancer dataset, Wilcoxon signed-rank test on common lncRNAs within a pathway was conducted, controlling FDR at 5%. To assess the statistical significance of the risk lncRNA expression patterns in cancer risk pathways of each cancer dataset, 1000 permutation tests were carried out. Cancer risk lncRNAs were randomly selected while preserving the pathway sizes for each permutation test. The significance of Wilcoxon signed-rank test for each pathway was re-calculated. Here, lncRNAs (FDR < 0.05) were defined as risk lncRNAs.

### Evaluation of the classification performance of cancer risk lncRNAs

To assess the classification performance of tumor and normal samples, a Support Vector Machine (SVM) was used while cancer risk lncRNAs expression values as classification features. For each cancer dataset that had been split into five parts, five-fold cross-validation was applied while the model is trained on the training set and tested on the testing set. In this manner, each part has been tested once. Then a receiver operating characteristic (ROC) curve was adopted to estimate classification performance. The area under the curve (AUC) value implied the classification performance^43^.

### Functional enrichment analysis

The hypergeometric test was applied to dissect the association between genes annotated to hallmark-associated GO terms and genes within these pathways in order to investigate the probable biological roles of cancer risk pathways. The probability of genes within a hallmarks related GO term for a cancer risk pathway i was calculated as:


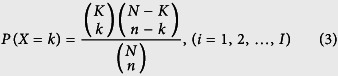


where N is the number of all genes in pathway i and a hallmark-associated GO term, n is the number of genes within a hallmark-associated GO term, K represents the number of genes in pathway i, k is the number of common genes annotated in a hallmark-associated GO term and pathway i and I is the total number of pathways. For each pathway, pathways (FDR < 0.05) were considered as significantly enriched pathways with genes which annotated to hallmark-associated GO terms.

### Survival analysis of cancer risk lncRNAs

The survival difference in overall survival (OS) between high-risk group and low-risk group was assessed by Kaplan-Meier plots, and the significance level was calculated by the univariate Cox regression and log-rank test.

## Additional Information

**How to cite this article**: Li, Y. *et al*. Identification of cancer risk lncRNAs and cancer risk pathways regulated by cancer risk lncRNAs based on genome sequencing data in human cancers. *Sci. Rep.* **6**, 39294; doi: 10.1038/srep39294 (2016).

**Publisher's note:** Springer Nature remains neutral with regard to jurisdictional claims in published maps and institutional affiliations.

## Supplementary Material

Supplementary Figure

Supplementary Dataset 1

Supplementary Dataset 2

Supplementary Dataset 3

Supplementary Dataset 4

Supplementary Dataset 5
