# Supplementary material for: Identification of cancer risk lncRNAs and cancer risk pathways regulated by cancer risk lncRNAs based on genome sequencing data in human cancers
Source: Sci Rep. 2016 Dec 19;6:39294. doi: 10.1038/srep39294 (PMC5171637; doi:10.1038/srep39294)
Supplement: Supplementary Figure [file srep39294-s1.pdf]

Identification of cancer risk lncRNAs and cancer risk pathways regulated by cancer risk lncRNAs based on genome sequencing data in human cancers

Yiran Li[1,†], Wan Li[1,†] , Binhua Liang[2,] , Liansheng Li[1], Li Wang[1], Hao Huang[1], Shanshan Guo[1], Yahui Wang[1], Yuehan He[1], Lina Chen[1,]* ,Weiming He[3,]*

[1]College of Bioinformatics Science and Technology, Harbin Medical University, Harbin, Hei Longjiang Province, China Postal code:150081

[2]National Microbology Laboratory, Public Health Agency of Canada, Winnipeg, Manitoba, Canada

[3]Institute of Opto-electronics, Harbin Institute of Technology, Harbin, Heilongjiang Province, China Postal code:150081

†These authors contributed equally to this work.

*Corresponding author: Lina Chen, email: chenlina@ems.hrbmu.edu.cn

Weiming He, email: hewm@hit.edu.cn

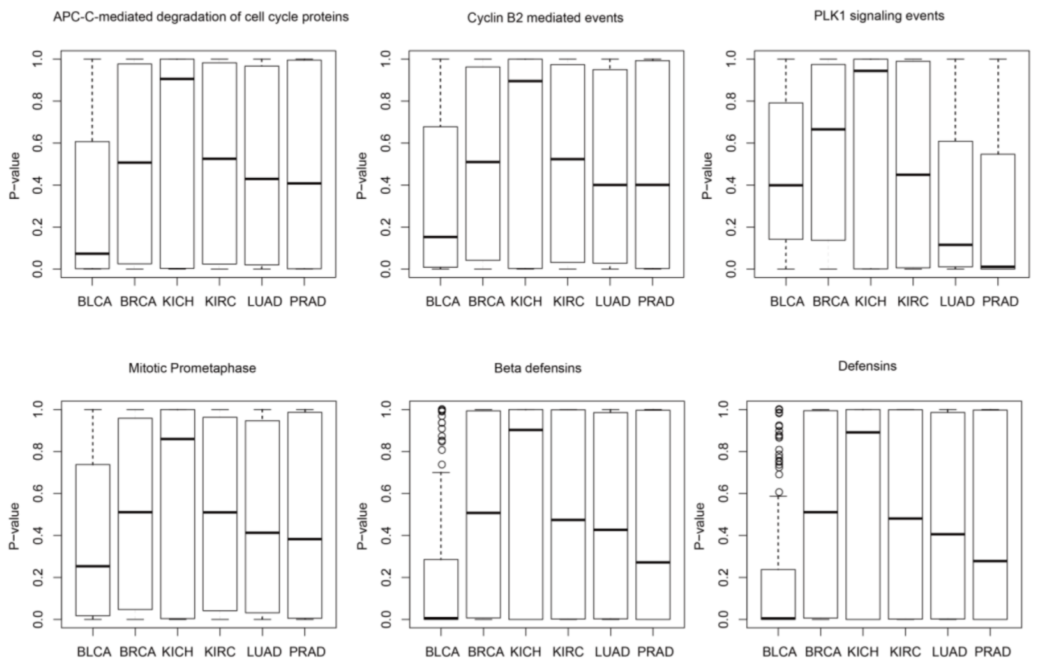
APC-C-mediated degradation of cell cycle proteins
Cyclin B2 mediated events
PLK1 signaling events
Mitotic Prometaphase
Beta defensins
Defensins
P-value
BLCA BRCA KICH KIRC LUAD PRAD

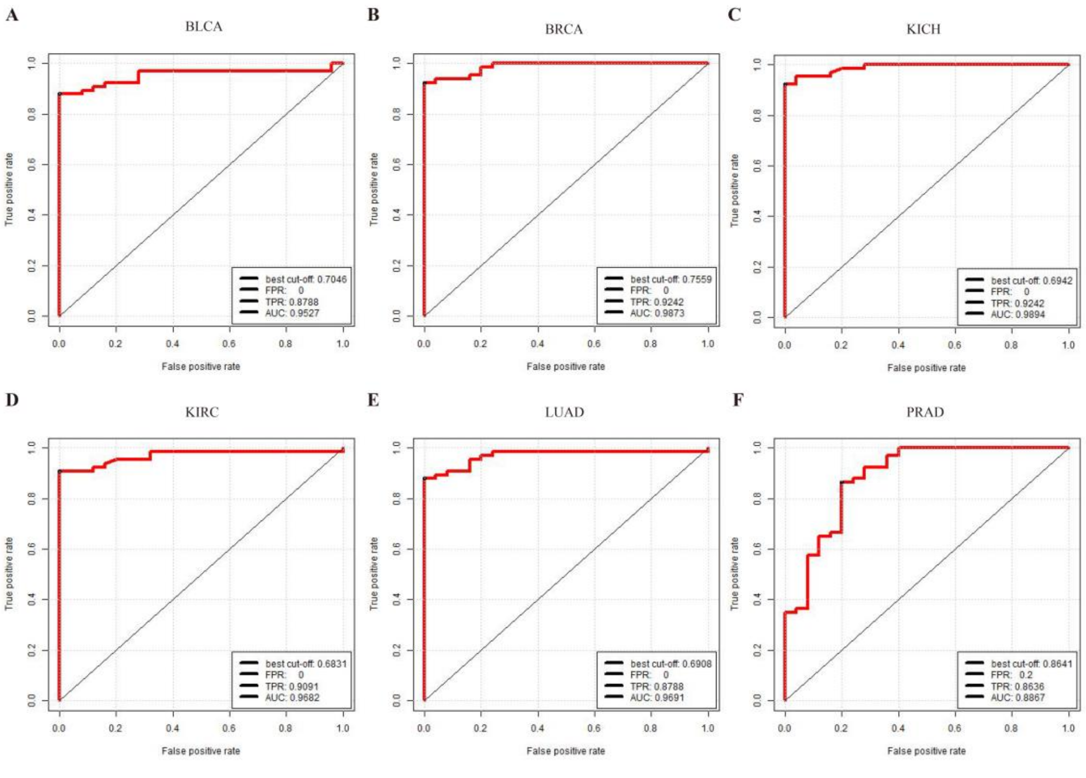
A
BLCA
best cut-off: 0.7046
FPR: 0
TPR: 0.8788
AUC: 0.9527
B
BRCA
best cut-off: 0.7559
FPR: 0
TPR: 0.9242
AUC: 0.9873
C
KICH
best cut-off: 0.6942
FPR: 0
TPR: 0.9242
AUC: 0.9894
D
KIRC
best cut-off: 0.6831
FPR: 0
TPR: 0.9091
AUC: 0.9682
E
LUAD
best cut-off: 0.6908
FPR: 0
TPR: 0.8788
AUC: 0.9691
F
PRAD
best cut-off: 0.8641
FPR: 0.2
TPR: 0.8636
AUC: 0.8867
True positive rate
False positive rate

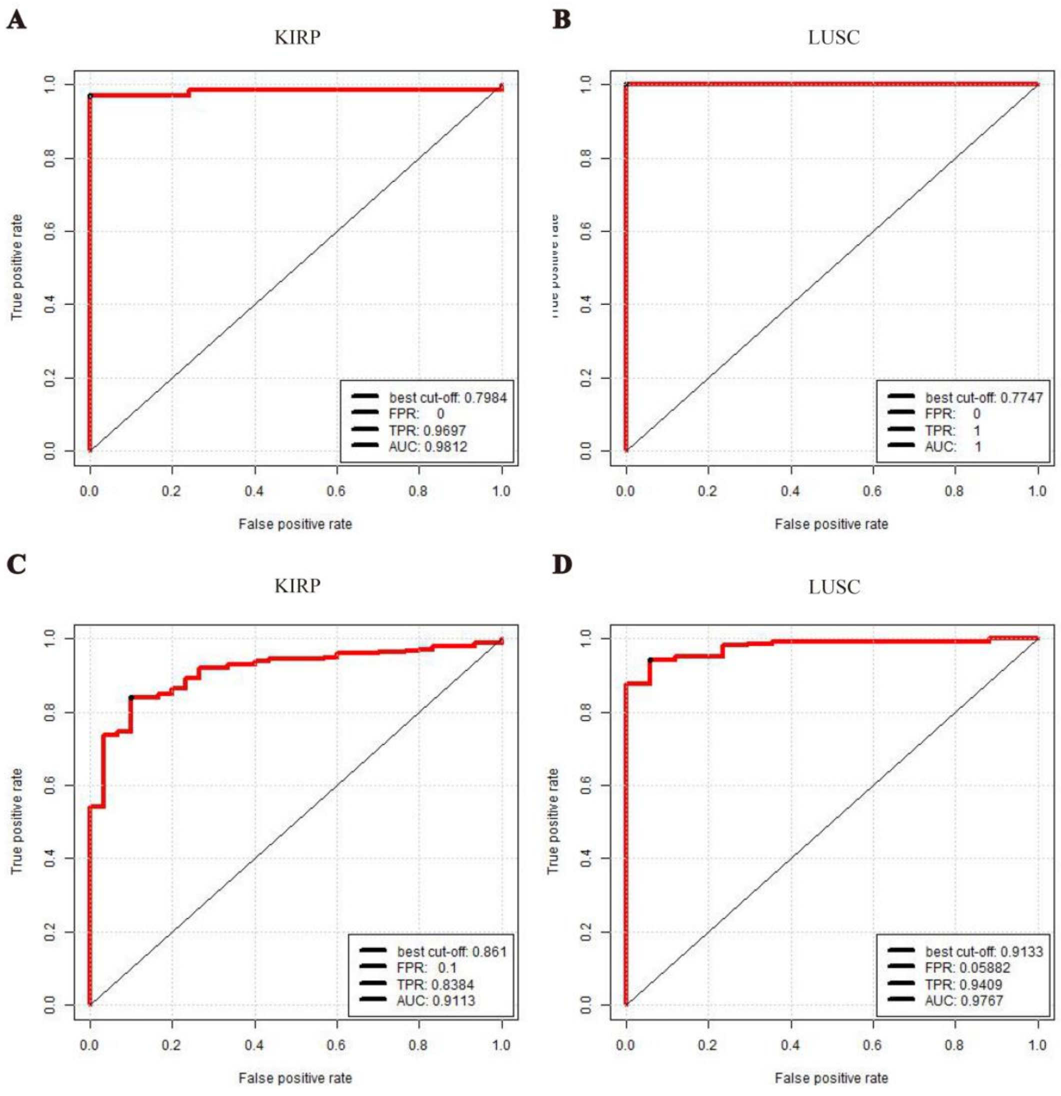
A
KIRP
True positive rate
False positive rate
best cut-off: 0.7984
FPR: 0
TPR: 0.9697
AUC: 0.9812
B
LUSC
best cut-off: 0.7747
FPR: 0
TPR: 1
AUC: 1
C
KIRP
best cut-off: 0.861
FPR: 0.1
TPR: 0.8384
AUC: 0.9113
D
LUSC
best cut-off: 0.9133
FPR: 0.05882
TPR: 0.9409
AUC: 0.9767

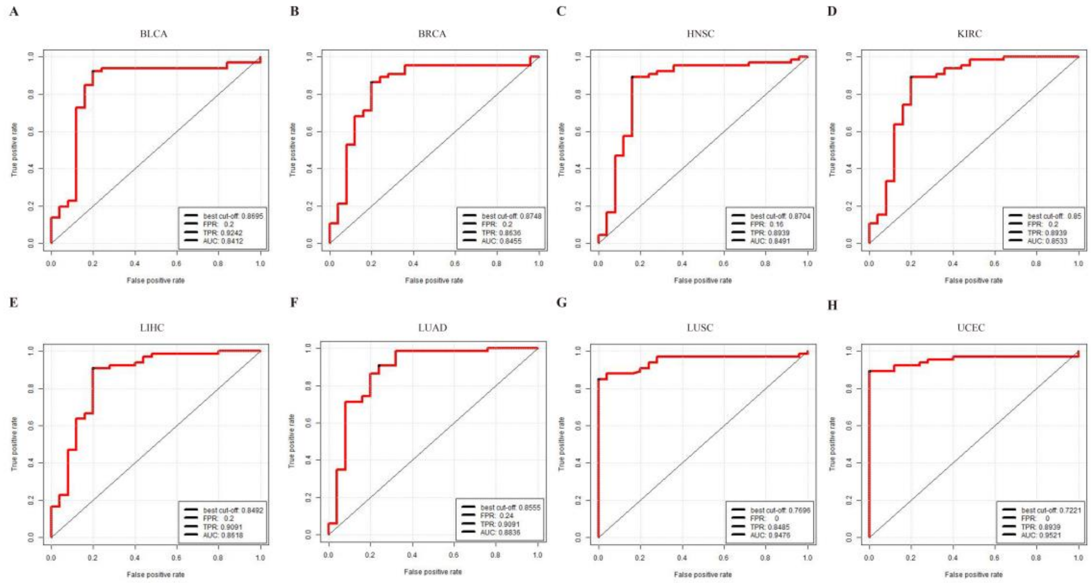
A
BLCA
best cut-off: 0.8695
FPR: 0.2
TPR: 0.9242
AUC: 0.8412
B
BRCA
best cut-off: 0.8748
FPR: 0.2
TPR: 0.8636
AUC: 0.8455
C
HNSC
best cut-off: 0.8704
FPR: 0.16
TPR: 0.8939
AUC: 0.8491
D
KIRC
best cut-off: 0.85
FPR: 0.2
TPR: 0.8939
AUC: 0.8533
E
LIHC
best cut-off: 0.8492
FPR: 0.2
TPR: 0.9091
AUC: 0.8618
F
LUAD
best cut-off: 0.8555
FPR: 0.24
TPR: 0.9091
AUC: 0.8836
G
LUSC
best cut-off: 0.7696
FPR: 0
TPR: 0.8485
AUC: 0.9476
H
UCEC
best cut-off: 0.7221
FPR: 0
TPR: 0.8939
AUC: 0.9521
True positive rate
False positive rate

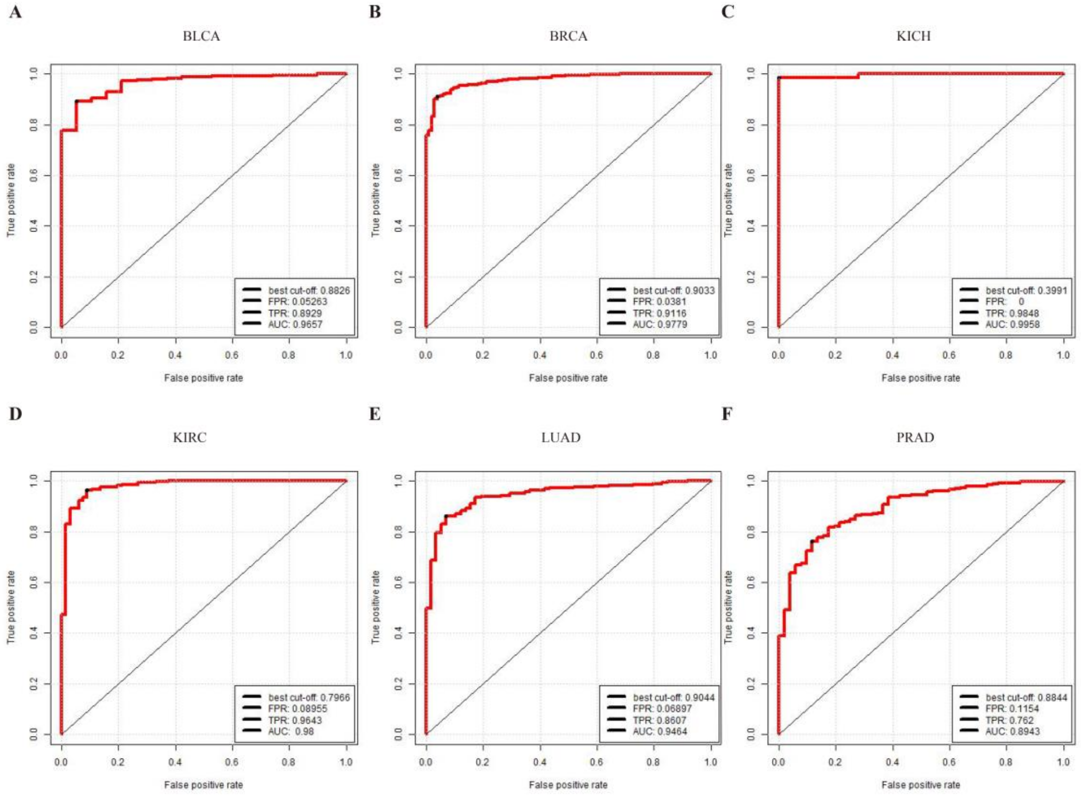
A
BLCA
True positive rate
False positive rate
best cut-off: 0.8826
FPR: 0.05263
TPR: 0.8929
AUC: 0.9657
B
BRCA
best cut-off: 0.9033
FPR: 0.0381
TPR: 0.9116
AUC: 0.9779
C
KICH
best cut-off: 0.3991
FPR: 0
TPR: 0.9848
AUC: 0.9958
D
KIRC
best cut-off: 0.7966
FPR: 0.08955
TPR: 0.9643
AUC: 0.98
E
LUAD
best cut-off: 0.9044
FPR: 0.06897
TPR: 0.8607
AUC: 0.9464
F
PRAD
best cut-off: 0.8844
FPR: 0.1154
TPR: 0.762
AUC: 0.8943

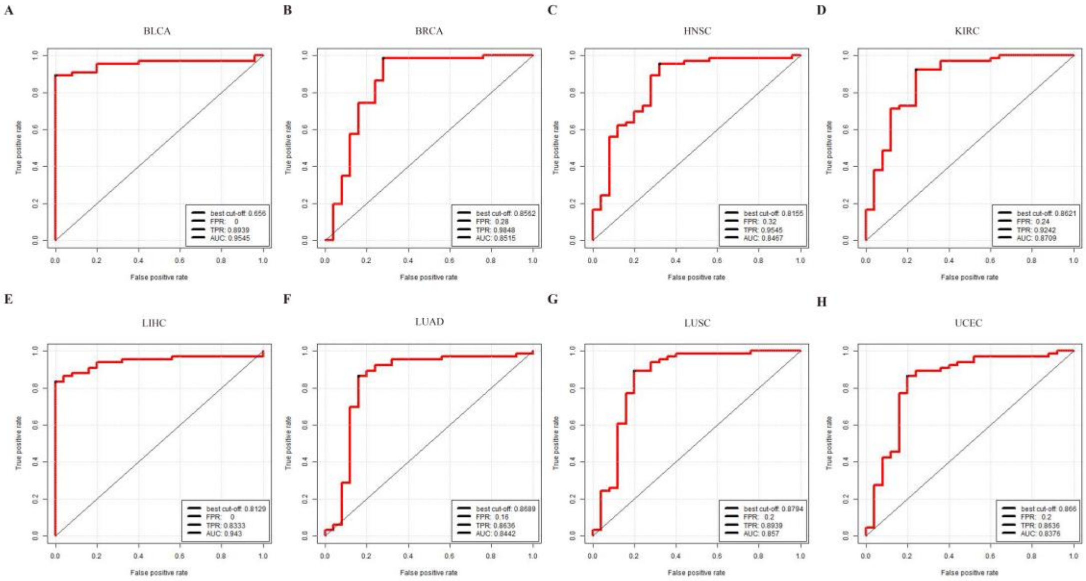
A
BLCA
True positive rate
False positive rate
best cut-off: 0.656
FPR: 0
TPR: 0.8939
AUC: 0.9545
B
BRCA
best cut-off: 0.8562
FPR: 0.28
TPR: 0.9848
AUC: 0.8515
C
HNSC
best cut-off: 0.8155
FPR: 0.32
TPR: 0.9545
AUC: 0.8467
D
KIRC
best cut-off: 0.8621
FPR: 0.24
TPR: 0.9242
AUC: 0.8709
E
LIHC
best cut-off: 0.8129
FPR: 0
TPR: 0.8333
AUC: 0.943
F
LUAD
best cut-off: 0.8689
FPR: 0.16
TPR: 0.8636
AUC: 0.8442
G
LUSC
best cut-off: 0.8794
FPR: 0.2
TPR: 0.8939
AUC: 0.857
H
UCEC
best cut-off: 0.866
FPR: 0.2
TPR: 0.8636
AUC: 0.8376
